# Length of phone use and glioma risk: a Mendelian randomization study

**DOI:** 10.1097/JS9.0000000000001563

**Published:** 2024-05-20

**Authors:** Qiang He, Wenjing Wang, GLIOGENE Consortium, Junpeng Ma

**Affiliations:** a Department of Neurosurgery, West China Hospital, Sichuan University; b Department of Pharmacy, Institute of Metabolic Diseases and Pharmacotherapy, West China Hospital, Sichuan University, Wuhou District, Chengdu, People’s Republic of China

Highlights

- Causal relationship between phone use and glioma risk remains inconclusive.
- We explore a Mendelian randomization analysis about the genetic relationship between the length of phone use and glioma and its subtype development.
- The length of phone use might increase the risk of overall glioma and low-grade glioma.

*Dear Editor,*

At present, the research on the treatment of glioma has achieved substantial progress. A meta-analysis comparing subtotal resection with gross total resection conducted by Wei Hua and his colleagues at the *Int J Surg* found the superior efficacy of GTR on survival^1^. However, the etiologic study on glioma and its subtypes is limited. In addition, the relationship between phone use and glioma risk remains inconclusive. Some studies supported that the glioma risk was increased by mobile phone^2^, while no associations have been reported^3^. Thus, if phone use as a potential risk factor is causally associated with glioma still needs further investigation.

Theoretically, findings from randomized controlled trials (RCTs) provide the highest level of evidence for establishing a causal relationship between the length of phone use and glioma risk. However, conducting an RCT to clarify this causal association is not feasible. Mendelian randomization (MR) is an epidemiological method that utilizes genetic variants to investigate causal relationships between exposures and glioma. This method is widely employed for making causal inferences, as it helps mitigate confounding factors and reverse causal biases often associated with small sample sizes and cross-sectional designs^4^. Here, this two-sample MR study aims to uncover a potential association between the length of phone use and glioma risk using the summary statistics of genome-wide association study (GWAS).

Summary statistics of length of phone use were obtained from UK Biobank in which 456 972 individuals were included (Supplementary Table S1, Supplemental Digital Content 1, http://links.lww.com/JS9/C581). The summary-level glioma datasets were sourced from a recent meta-analysis involving eight glioma GWAS^5^. This encompassed a cohort of 12 488 cases and 18 169 controls (Supplementary Table S2, Supplemental Digital Content 1, http://links.lww.com/JS9/C581). The classification of gliomas was based on malignancy grade, distinguishing between pilocytic astrocytoma WHO grade I, diffuse ‘low-grade’ glioma (WHO grade II), anaplastic glioma (WHO grade III), and GBM (WHO grade IV). Further categorization included low-grade glioma (LGG, non-GBM, *N*=5820) and GBM (*N*=6183). All participants were European. To qualify as valid instrumental variables, genetic variants must meet three assumptions: (1) IVs are significantly associated with exposure; (2) IVs are not related to any confounders of the exposure-outcome association; and (3) IVs affect the outcome only via the exposure. To satisfy these assumptions, the genetic instruments for exposure were included at a genome-wide significance level (*P*<5×10^-8^). On the European population as a reference, a clumping window (10 000 kb) and an r^2^<0.001 are used to select the SNPs. The strength of the MR analysis was assessed using the F-statistics=(Bets/Se)^2^. SNPs with F-statistics less than 10 were excluded. We also used the PhenoScanner V2 (http://www.phenoscanner.medschl.cam.ac.uk/) to remove the confoundering SNP (Supplementary Table S3, Supplemental Digital Content 1, http://links.lww.com/JS9/C581). Then, six methods were applied to investigate the effects of exposure on glioma, including inverse variance weighted (IVW), maximum likelihood (ML), Weighted median, MR-Egger, Pleiotropy residual sum, and outlier (MR-PRESSO), and robust adjusted profile score (MR.RAPS). The random-effects IVW method was applied as the primary method. In addition, Bonferroni correction with a threshold *P*-value of 0.0166 (0.05/3) was implemented. Cochran’s *Q* test was employed to assess the heterogeneity. MR-Egger and MR-PRESSO global tests were applied to identify potential horizontal pleiotropy. The strength of the SNPs was evaluated using F-statistics, with a value greater than 10 indicating potential bias. Additionally, we implemented Steiger filtering to verify the directionality of the associations. All statistical analyses were performed using R version 4.3.0 (R Foundation for Statistical Computing).

In this two-sample MR, all the F-statistics exceed 10. The genetic variants were displayed in Supplementary Table S4 (Supplemental Digital Content 1, http://links.lww.com/JS9/C581). The length of phone use had causal effects on glioma. Significant risk for glioma and LGG has been increased by long phone use (glioma, OR=1.73, 95% CI=1.19–2.51, *P*=0.0037; LGG, OR=2.81, 95% CI=1.84–4.30, *P*=1.73e-06, Fig. 1). No causal effects on GBM were detected (*P*=0.3763). Other methods supported effect sizes and direction. No heterogeneity and pleiotropy were identified by Cochran’s *Q* test and global test (All *P*>0.05, Supplementary Table S5, Supplemental Digital Content 1, http://links.lww.com/JS9/C581). Steiger filtering confirmed directionality.

**Figure 1:**
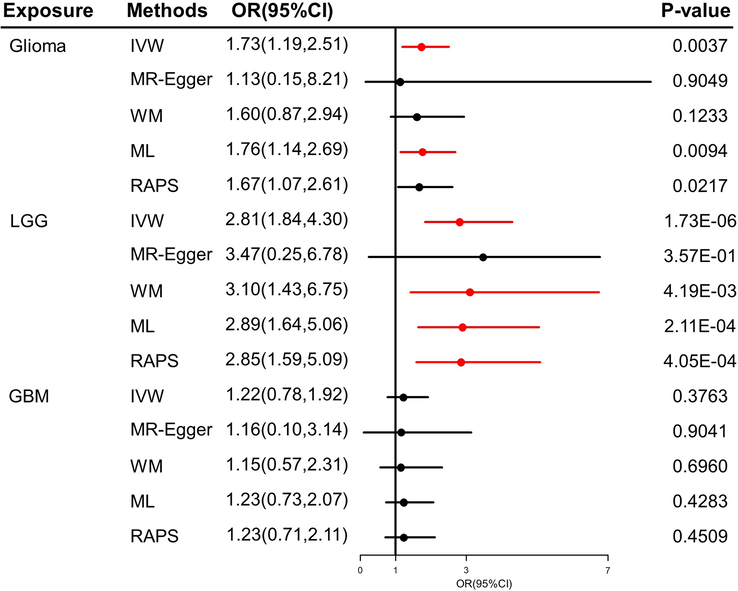
The MR results of the causal association between length of phone use and glioma and its subtypes. GBM, glioblastoma; IVW, inverse variance weighted; LGG, low-grade glioma; ML, maximum likelihood; MR, Mendelian randomization; OR, odds ratio; RAPS, robust adjusted profile score; WM, weighted median.

Several limitations are acknowledged. Firstly, the reliance on summary-level data from individuals of European ancestry may restrict the generalizability of the findings to other populations. Secondly, the inability to access individual-level genetic data limits the exploration of the relationship at a more granular level. Lastly, the absence of GWAS datasets that incorporate molecular subtyping for glioma precludes the investigation of their causal relationship. Future endeavors are required in multiancestry, molecular subtyping, and individual-level relationship to validate and expand he current findings.

## Ethical approval

All named authors meet the ICMJE criteria for authorship in this article, take responsibility for the integrity of the work as a whole, and have given their approval for this version to be published.

## Consent

The summary-level statistics for both exposure and outcome datasets were derived from de-identified datasets of previously approved studies. Necessary ethics committee approvals and participant informed consent have been secured. This MR study is exempt from ethical approval, given its exclusive use of summary-level datasets.

## Sources of funding

Not applicable.

## Author contribution

Q.H. and J.W.: analyzed and interpreted the data and wrote the manuscript; J.M.: designed the study and reviewed the manuscript.

## Conflicts of interest disclosure

All authors report no conflicts of interest in this work.

## Research registration unique identifying number (UIN)

Not applicable.

## Guarantor

All authors.

## Data availability statement

Summary statistics from the glioma GWAS meta-analysis are available from the European Genome-phenome Archive (EGA, http://www.ebi.ac.uk/ega/).

## Provenance and peer review

My paper was not invited.

## Supplementary Material

## GLIOGENE Consortium

Christopher I Amos, Jill S Barnholtz-Sloan, Jonine L Bernstein, Melissa L Bondy, Elizabeth B Claus, Richard S Houlston, Dora Il’yasova, Robert B Jenkins, Christoffer Johansen, Daniel Lachance, Rose Lai, Beatrice S Melin, Ryan T Merrell, Sara H Olson, Siegal Sadetzki, Joellen Schildkraut, Sanjay Shete.
