# Supplementary material for: Length of phone use and glioma risk: a Mendelian randomization study
Source: Int J Surg. 2024 May 20;110(8):5254–5. doi: 10.1097/JS9.0000000000001563 (PMC11326033; doi:10.1097/JS9.0000000000001563)
Supplement: Supplementary file 1 [file js9-110-5254-s001.docx]

**Table S1** The summary-level GWAS data sources in MR analysis

**Table S2** The summary-level GWAS data sources for glioma in MR analysis

**Table S3** The removed SNP through PhenoScanner

**Table S4** The genetic instruments of length of phone use in MR analysis

**Table S5** The sensitivity analysis of length of phone use on glioma

**Table S1** The summary-level GWAS data sources in MR analysis

| **Traits** | **PMID** | **Cases** | **Controls** | **Sample size** | **Female** | **Race** | **Web OR Consortium** |
| --- | --- | --- | --- | --- | --- | --- | --- |
| **Exposure** |  |  |  |  |  |  |  |
| Phone length | - | - | - | 456,972 | - | European | https://www.ukbiobank.ac.uk/ |
| **Outcome** |  |  |  |  |  |  |  |
| Glioma | 28346443 | 12488 | 18169 | 30657 | NA | European | GliomaScan Consortium |
| LGG | 28346443 | 5820 | 18169 | 23989 | NA | European | GliomaScan Consortium |
| GBM | 28346443 | 6183 | 18169 | 24352 | NA | European | GliomaScan Consortium |

GWAS, genome wide association study; MR, Mendelian randomization; CSF, cerebrospinal fluid; LGG, low-grade glioma; GBM, glioblastoma

**Table S2** The summary-level GWAS data sources for glioma in MR analysis

| **Dataset** | **Pubmed ID** | **LGG cases** | **GBM cases** | **All-glioma cases** | **Number of controls** |
| --- | --- | --- | --- | --- | --- |
| UK-GWAS | 17636416 | 361 | 270 | 631 | 2699 |
| French-GWAS | 21531791 | 993 | 430 | 1423 | 1190 |
| German-GWAS | 26424050 | 415 | 431 | 846 | 1310 |
| MDA-GWAS | 19578367 | 523 | 652 | 1175 | 2236 |
| UCSF- SFAGS | 19578367 | 166 | 511 | 677 | 3940 |
| GliomaScan | 22886559 | 472 | 903 | 1653 | 2725 |
| GICC | 26656478 | 1898 | 2460 | 4564 | 3265 |
| UCSF/Mayo | 19578366 | 992 | 526 | 1519 | 804 |
| **Total** |  | **5820** | **6183** | **12488** | **18169** |

GWAS, gene-wide association study; MR, mendelian randomization; GBM, glioblastoma; LGG, low-grade glioma

**Table S3** The removed SNP through PhenoScanner

| **rsid** | **trait** | **chr** | **pos** | **p** | **Pumbed ID** |
| --- | --- | --- | --- | --- | --- |
| rs10107145 | systolic blood pressure | 8 | 10900703 | 1.00E-08 | 28739976 |
| rs10107145 | systolic blood pressure | 8 | 10900703 | 1.00E-17 | 28739976 |
| rs77878475 | smoking initiation | 16 | 17964691 | 3.00E-10 | 30679032 |
| rs10828247 | waist-hip ratio | 10 | 21533927 | 2.00E-12 | 30239722 |
| rs77878475 | smoking behavior | 16 | 17964691 | 3.00E-11 | 30643258 |
| rs6131703 | risk-taking behaviour | 20 | 15774039 | 4.00E-12 | 30643258 |
| rs9896202 | skin aging measurement | 17 | 79804428 | 3.00E-08 | 32339537 |
| rs77878475 | smoking status measurement | 16 | 17964691 | 2.00E-08 | 30595370 |
| rs10828247 | cancer | 10 | 21533927 | 3.00E-13 | 32887889 |
| rs10828247 | breast carcinoma | 10 | 21533927 | 3.00E-13 | 32887889 |
| rs10828247 | brain measurement | 10 | 21533927 | 1.00E-82 | 34910505 |
| rs10828247 | migraine disorder | 10 | 21533927 | 8.00E-09 | 35115687 |
| rs77878475 | risk-taking behaviour | 16 | 17964691 | 1.00E-09 | 36324656 |
| rs9896202 | neuroimaging measurement | 17 | 79804428 | 3.00E-11 | 35164939 |

**Table S4** The genetic instruments of length of phone use in MR analysis

| **SNP** | **chr** | **effect_allele** | **other_allele** | **pval** | **se** | **beta** | **eaf** |
| --- | --- | --- | --- | --- | --- | --- | --- |
| rs12145998 | 1 | T | C | 2.90E-09 | 0.0032 | -0.0193 | 0.2656 |
| rs1892417 | 1 | C | T | 1.30E-14 | 0.0034 | 0.0262 | 0.2287 |
| rs344868 | 2 | T | C | 3.20E-08 | 0.0033 | 0.0182 | 0.2467 |
| rs6718176 | 2 | G | C | 1.70E-10 | 0.0028 | -0.0182 | 0.5129 |
| rs359265 | 2 | A | G | 6.40E-13 | 0.0029 | 0.0210 | 0.6068 |
| rs11682846 | 2 | T | C | 9.90E-10 | 0.0029 | -0.0175 | 0.4851 |
| rs849527 | 2 | G | A | 1.50E-08 | 0.0029 | -0.0163 | 0.5466 |
| rs6780051 | 3 | T | G | 6.90E-11 | 0.0061 | 0.0397 | 0.0580 |
| rs1512142 | 4 | A | G | 8.30E-09 | 0.0029 | -0.0165 | 0.4429 |
| rs17374152 | 5 | G | A | 5.30E-09 | 0.0033 | -0.0195 | 0.2386 |
| rs17156711 | 5 | G | A | 4.10E-09 | 0.0031 | -0.0183 | 0.2986 |
| rs2161220 | 5 | A | G | 4.00E-10 | 0.0033 | 0.0207 | 0.2478 |
| rs78166132 | 5 | C | T | 4.90E-10 | 0.0049 | -0.0305 | 0.0935 |
| rs10807124 | 6 | A | G | 3.10E-08 | 0.0032 | -0.0176 | 0.2743 |
| rs28713780 | 7 | C | T | 1.10E-08 | 0.0030 | -0.0169 | 0.6413 |
| rs13266457 | 8 | T | C | 1.70E-08 | 0.0030 | -0.0171 | 0.3295 |
| rs7859831 | 9 | T | C | 2.10E-08 | 0.0041 | -0.0231 | 0.1392 |
| rs853946 | 10 | T | C | 1.80E-08 | 0.0028 | 0.0160 | 0.4682 |
| rs1320650 | 11 | A | T | 7.20E-09 | 0.0029 | 0.0171 | 0.3669 |
| rs11236714 | 11 | T | C | 1.80E-08 | 0.0036 | -0.0201 | 0.1961 |
| rs11229008 | 11 | A | G | 3.90E-08 | 0.0059 | -0.0327 | 0.0626 |
| rs8014346 | 14 | A | G | 3.70E-11 | 0.0028 | 0.0188 | 0.5347 |
| rs12437348 | 14 | A | G | 4.20E-08 | 0.0031 | 0.0172 | 0.7100 |
| rs11655813 | 17 | T | C | 1.20E-09 | 0.0030 | 0.0180 | 0.3550 |
| rs6063374 | 20 | G | A | 1.00E-17 | 0.0034 | 0.0294 | 0.7806 |
| rs2836920 | 21 | G | T | 2.30E-10 | 0.0029 | 0.0186 | 0.3955 |

MR, Mendelian randomization

**Table S5** The sensitivity analysis of length of phone use on glioma

| outcome | exposure | Methods | F-statistic | R2 | Egger_intercept | P-Egger_intercept | Causal_direction | Steiger’s *P* | Cochran’s | Cochran’s *P* |
| --- | --- | --- | --- | --- | --- | --- | --- | --- | --- | --- |
| Glioma | PhoneLength | IVW | 38.33 | 0.0021 | 0.0087 | 0.6702 | TRUE | 0.0019 | 17.85 | 0.7652 |
|  |  | MR-Egger |  |  |  |  |  |  | 17.67 | 0.7253 |
| LGG | PhoneLength | IVW | 38.33 | 0.0021 | -0.0043 | 0.8721 | TRUE | 0.0414 | 13.56 | 0.9385 |
|  |  | MR-Egger |  |  |  |  |  |  | 13.54 | 0.9168 |
| GBM | PhoneLength | IVW | 38.33 | 0.0021 | 0.0010 | 0.9653 | TRUE | 0.0038 | 17.63 | 0.7770 |
|  |  | MR-Egger |  |  |  |  |  |  | 17.63 | 0.7276 |


MR, Mendelian randomization
